# Evaluation of Bacterial Expansin EXLX1 as a Cellulase Synergist for the Saccharification of Lignocellulosic Agro-Industrial Wastes

**DOI:** 10.1371/journal.pone.0075022

**Published:** 2013-09-23

**Authors:** Hui Lin, Qi Shen, Ju-Mei Zhan, Qun Wang, Yu-Hua Zhao

**Affiliations:** 1 Institute of Microbiology, College of Life Sciences, Zhejiang University, Hangzhou, China; 2 Institute of Plant Science, College of Life Sciences, Zhejiang University, Hangzhou, China; 3 Institute of Environment, Resource, Soil and Fertilizer, Zhejiang Academy of Agricultural Sciences, Hangzhou, China; California State University Fullerton, United States of America

## Abstract

Various types of lignocellulosic wastes extensively used in biofuel production were provided to assess the potential of EXLX1 as a cellulase synergist. Enzymatic hydrolysis of natural wheat straw showed that all the treatments using mixtures of cellulase and an optimized amount of EXLX1, released greater quantities of sugars than those using cellulase alone, regardless of cellulase dosage and incubation time. EXLX1 exhibited different synergism and binding characteristics for different wastes, but this can be related to their lignocellulosic components. The cellulose proportion could be one of the important factors. However, when the cellulose proportion of different biomass samples exhibited no remarkable differences, a higher synergism of EXLX1 is prone to occur on these materials, with a high proportion of hemicellulose and a low proportion of lignin. The information could be favorable to assess whether EXLX1 is effective as a cellulase synergist for the hydrolysis of the used materials. Binding assay experiments further suggested that EXLX1 bound preferentially to alkali pretreated materials, as opposed to acid pretreated materials under the assay condition and the binding preference would be affected by incubation temperature.

## Introduction

Lignocellulosic waste is a promising resource for producing fuels and chemicals, both natural and man-made [1]. As the most abundant and renewable source on earth, lignocellulose consists of three major components: cellulose, hemicellulose and lignin [2]. Deconstruction of lignocellulose into fermentable sugars is a key process in its conversion to high-value chemicals and an array of glycoside hydrolases is required. There is no single enzyme that is able to hydrolyze lignocellulose biomass efficiently, due to its tightly-packed structure and complex components. Design of glycoside hydrolase mixtures that function synergistically to release sugars from biomass has been known to be an effective strategy [3], [4]. Recently, combined utilization of proteins lacking glycoside hydrolase activity (non-GH) with glycoside hydrolases such as cellulase has been suggested as another effective option to facilitate the release of sugars from lignocellulosic biomass [5], [6].

Expansins and expansin-like proteins are one kind of non-GH proteins that do not directly hydrolyze lignocellulose but can increase the hydrolysis efficiency of glycoside hydrolases in a synergistic manner [6], [7], [8]. The binding and loosening functions of expansin to cell wall components imply that it disrupts the hydrogen bonds in CPs and enhances the accessibility of cell wall degrading enzymes [9], [10]. Many expansins or expansin-like proteins, such as LOOS1 [11], swollenin [12], AfSwo1 [13] and maize β-expansin [7], have been found that can enhance the activity of glycoside hydrolases in the saccharification of plant biomass. Quiroz-Castaneda et al. [11] showed that *Agave tequilana* fiber, extensively grown in some areas of Mexico, can be a susceptible substrate for a cocktail of commercial cellulases and xylanases in the presence of LOOS1. In the study of Chen et al. [13], cellulase used together with the expansin AfSwo1 from *Aspergillus fumigatus* to hydrolyze the avicel resulted in a 13.2% increase in the sugar yield in comparison with cellulase used alone, even though the cellulase loading in these trials was high.

EXLX1 is an expansin-like protein encoded by the *yoaJ* gene of *Bacillus subtilis* that can be produced by *Escherichia coli* [6], [8], [14]. EXLX1 has received increasing attentions as a result of emerging reports such as the binding characterization, the cell wall-creeping study and the structure-function analysis [6], [14], [15]. However, the synergistic characteristics of EXLX1 have not been well characterized, especially using natural plant biomass as substrates [16]. In most previous reports, synergistic effects of EXLX1 were evaluated using pure cellulose such as avicel and filter paper and investigated with a low loading of cellulase [6], [16]. Actually, some reports have indicated that the weakening activity of expansin could be stronger in cellulose-xyloglucan composite materials than in cellulose only materials [15], [17], [18]. In this work, EXLX1 was characterized as a commercial cellulase synergist using various types of plant biomass wastes extensively used in biofuel production. The information provided would be useful for the evaluation of EXLX1 as a biochemical agent in cellulosic biomass conversion to reduce the cost of bioenergy production, although the mechanism of this process still needs further investigation.

## Materials and Methods

### Ethics Statement

All the materials used in this study such as Wheat straw collected from Fuyang city of Anhui province and Switchgrass obtained from Beijing City, were all agricultural waste widely distributed in the rural areas in China. As the materials collection activities had no conflicts of interest and environmental hazards, there were no specific permissions required. And the field studies did not involve endangered or protected species.

### Cloning, expression and purification of EXLX1

The gene encoding EXLX1 protein (Primary accession No. in Uni-ProtKB: O34918) was amplified from genomic DNA of *Bacillus subtilis* subsp. *Subtilis* strain 168 M (ATCC27370) by PCR using 5′ CGGAATTCGCATATGACGACCTGCATG 3′ and 5′ CCGCTCGAGTTCAGGAAACTGAACATGGC 3′ as primers. The *EXLX1* gene was cloned into pET21a (+) vector (Novagen) between the EcoRI and XhoI sites, followed by transformation into *Escherichia coli* BL21 (DE3-pLys) for expression.

The original signal peptide was removed from the recombinant EXLX1 and a 6-histidine tag was added at the carboxyl terminus (Table S1). The recombinant cells harboring pET21a (+)-EXLX1 were cultivated in Luria-Bertani medium (pH 7.0) supplemented with 100 µg ml^−1^ ampicillin. Cultures were grown to OD_600_≈0.5 at 37°C and then induced with 1 mM isopropyl β-D-1-thiogalactopyranoside (IPTG) for more than 5 h at 30°C. Cells were harvested by centrifugation at 12000 rpm for 10 min, washed twice with Buffer A (50 mM NaH_2_PO_4_, 500 mM NaCl, pH 8.0) and then disrupted by sonication. The cell debris was removed by centrifugation at 12000 rpm and 4°C for 30 min. The supernatant was loaded onto a column with Ni-NTA Agarose (Qiagen), which was pre-equilibrated with Buffer A. After binding, the column was subsequently washed with Buffer B (50 mM NaH_2_PO_4_, 500 mM NaCl, and 20 mM imidazole, pH 8.0) until no more protein was eluted. Finally, EXLX1 was eluted with Buffer C (50 mM NaH_2_PO_4_, 500 mM NaCl, and 250 mM imidazole, pH 8.0) and the eluted protein was stored in buffer D (50 mM NaH_2_PO_4_, 100 mM NaCl and 25% glycerol, pH 7.0) at −20°C after concentrated by ultrafiltration.

### Untreated (UNT) and pretreated plant biomass

Except for Wheat straw and Switchgrass, other biomass samples such as Rice straw, Green Reed, Sugarcane bagasse and Chinese fir sawdust (Wood) were all collected from local markets in Hangzhou. Dilute acid pretreatment (0.7% H_2_SO_4_, 121°C, 1 h, ratio of straw to liquid 10%) and alkaline peroxide pretreatment (2.5% H_2_O_2_, pH 11.5, 37°C, 24 h, ratio of straw to liquid 10%) was applied to biomass samples [19], respectively. Both alkaline peroxide pretreated (ALKALI) materials and dilute acid pretreated (ACID) materials were washed with distilled water till neutral pH was achieved. All the materials were dried before being milled to lower than 20 meshes. Compositional analysis [20] was further conducted on the UNT, ACID and ALKALI biomass samples.

### Enzymatic hydrolysis

Enzymatic hydrolysis was performed in a 1.5 ml microcentrifuge tube containing 5 mg of substrate (e.g. Whatman No. 1 filter paper, biomass samples) according to a previously published study with slight modifications [6]. Both the commercial cellulase (Celluclast 1.5L, Novozymes, Bagsvaerd, Denmark), EXLX1 and bovine serum albumin (BSA) were diluted to their corresponding concentrations in 750 µl final volume of citrate buffer (0.05 M, pH 4.8). The enzymatic hydrolysis was performed at 50°C before the reducing sugar (RS) was determined using dinitrosalicylic acid (DNS) reagent [21]. The synergetic activity of EXLX1 was calculated as described before [6]:
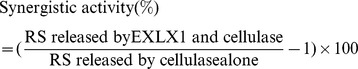



### Thermal- and pH-stability of EXLX1

The temporary thermal-stability of EXLX1 was determined by assaying the residual synergistic activity of EXLX1 after incubating EXLX1 at temperatures ranging from 25°C to 70°C for 30 min. The long-term thermal-stability was determined from the residual synergistic activity after incubating EXLX1 at temperatures ranging from 37°C to 60°C for 48 h. In order to determine the effect of pH on the stability of EXLX1, the synergistic activity was assayed after incubating EXLX1 at 4°C for 48 h with the following buffers: 100 mM citrate buffer for pH 3.0–6.0, 200 mM sodium phosphate buffer for pH 6.0–8.0, and 100 mM glycine-NaOH for pH 8.6–10.0. Enzymatic hydrolysis was incubated for 36 h using Whatman No. 1 filter paper as the substrate. The concentration of the EXLX1 and the cellulase in the reaction system was 200 µg/g substrate (0.5 µg) and 0.06 FPU/g substrate, respectively. The residual activity (%) of the pretreated EXLX1 was calculated according to:
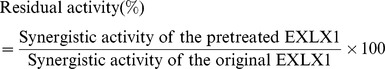



### Binding assay

All the UNT, ACID and ALKALI materials were provided as binding matrices. 30 µg of EXLX1 was mixed with 2.5 mg of binding matrix, respectively, suspended in 300 µl of citrate buffer (0.05 M, pH 4.8) and potassium phosphate buffer (0.05 M, pH 7.0). The mixture was incubated at 30°C for 4 h. After incubation, the supernatant was obtained by centrifuging at 12000 rpm for 5 min and subsequently quantified by the Bradford assay [22]. The amount of bound protein was determined by subtracting the amount of the unbound protein from the total amount of protein.

### Statistical analysis

All the above-mentioned assays were repeated more than four times. Statistical analyses such as variance analysis (ANOVA) and correlation analysis were all completed with SPSS 16.0 (Chicago, IL).

## Results and Discussion

### Production of bioactive EXLX1

**Figure 1:**
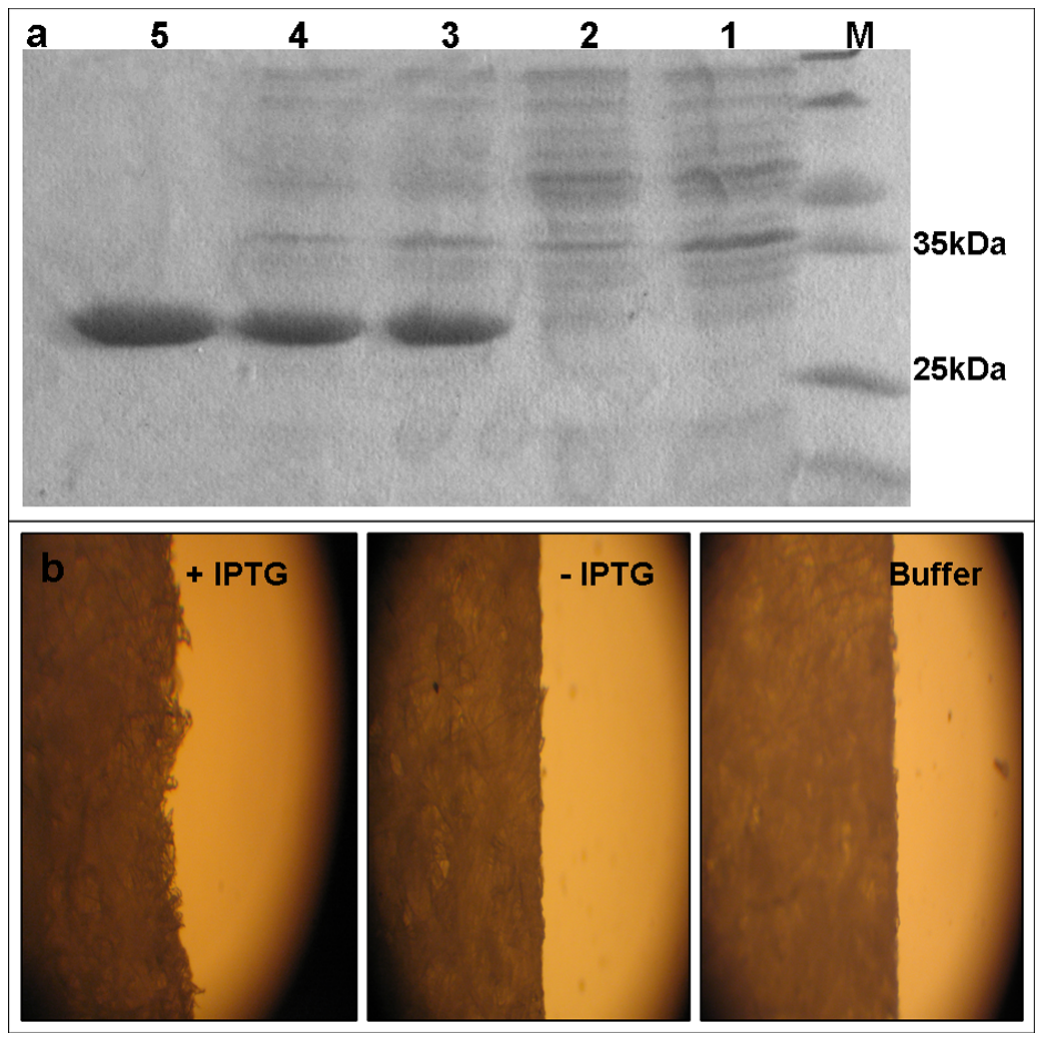
EXLX1 production, purification and filter paper disruption. (a) Sodium dodecyl sulfate polyacrylamide gel electrophoresis (SDS-PAGE) analysis: Lane M, protein molecular weight marker; Lane 1, the total cell lysate of the culture without IPTG; Lane 2, the soluble fraction of the culture without IPTG; Lane 3, the total cell lysate of the culture with IPTG; Lanes 4, the soluble fraction of the culture with IPTG; Lane 5, purified EXLX1 (about 26 kDa calculated based on amino acid sequences). (b) Light microscopy graphs of filter paper incubated with the soluble fraction of IPTG-induced culture, soluble fraction of the culture without IPTG and buffer A at 30°C for 12 h, respectively. Both the cell lysate and the soluble fraction were dissolved in buffer A (50 mM NaH_2_PO_4_, 500 mM NaCl, pH 8.0).

Recombinant EXLX1 was successfully expressed in *E. coli* and primarily exists in the soluble fraction (Fig.1a). As shown in Fig.1b, it could be found that the soluble fraction of IPTG induced cells exhibited remarkable disruptive activity against filter paper, which indicated the effective cell wall loosening of the recombinant EXLX1. Further, EXLX1 was purified using a 6-His tag located at the carboxyl-terminus of the protein (Fig.1a) and approximately 35 mg of purified EXLX1 was obtained from 1 L of culture broth. Determination of the synergistic activity of the purified EXLX1 with cellulase was performed with BSA used instead of EXLX1 in the negative control experiments. A synergistic activity (%) of 77.41±1.97 was obtained by incubating 5 mg filter paper with 0.5 µg of EXLX1 (200 µg/g substrate) and 0.06 FPU/g of cellulase for 36 h. The synergistic activity (%) was found to be 52.04±3.97 when the filter paper was incubated with 5 µg of EXLX1 (2000 µg/g substrate) and 0.6 FPU/g substrate of cellulase. Similar with the results published previously [6], BSA also exhibited a synergistic effect on cellulase activity but remarkably lower than EXLX1. The synergistic activity (%) was only 19.43±5.02 when 5 µg of BSA (2000 µg/g substrate) was incubated with 0.6 FPU/g substrate of cellulase for enzymatic hydrolysis. The above-mentioned results demonstrated that the recombinant EXLX1 in the present work exhibited similar synergism and loosening characteristics with those obtained in previous reports [6], [8], [14], although an additional polypeptide from the expression vector was involved at its amino terminus.

### Thermal- and pH-stability of EXLX1

**Figure 2:**
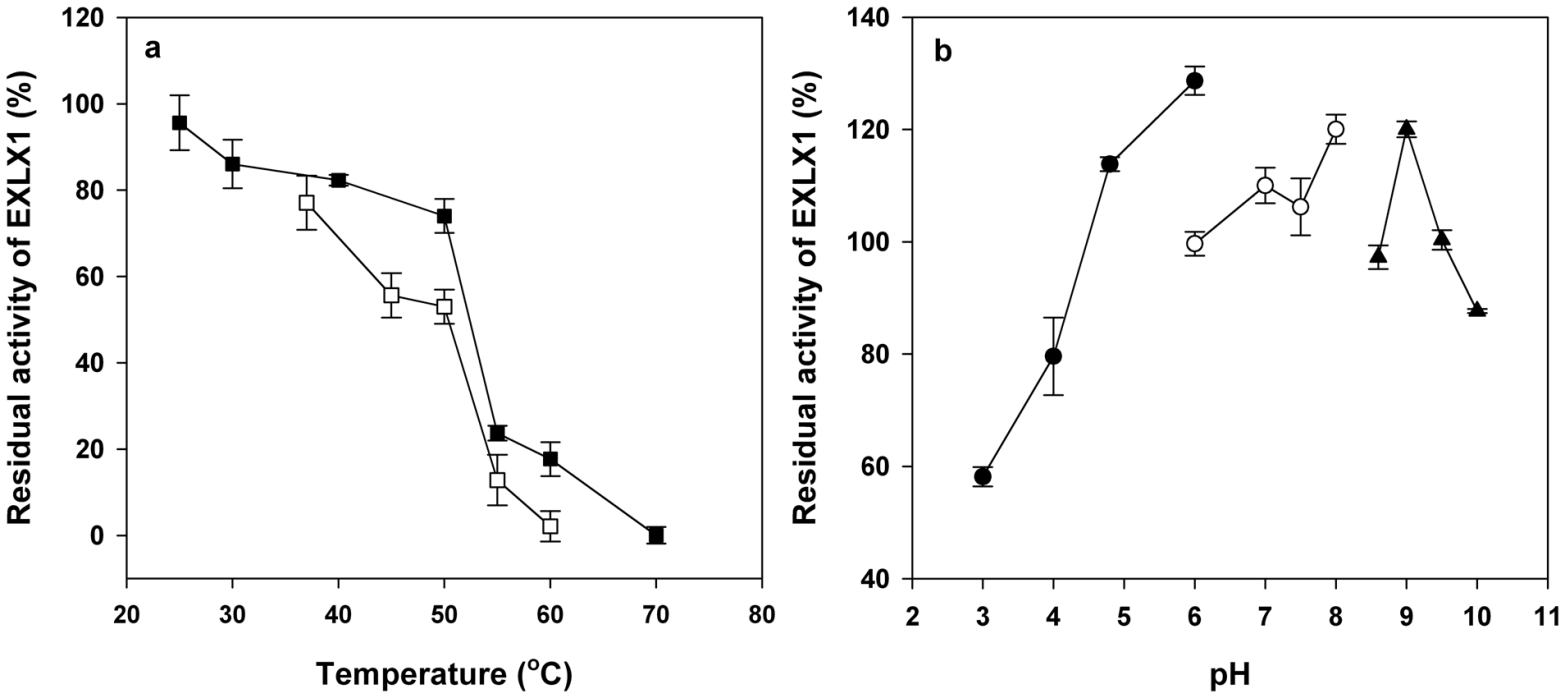
Thermal- and pH-stability of EXLX1. (a) Thermal-stability of EXLX1. EXLX1 was incubated at various temperatures for 30 min (black squares) and 48 h (open squares), respectively. The residual synergistic activity of the treated protein was then determined at 50°C after incubated for 36 h as described in Materials and Methods. (b) pH-stability of EXLX1. The protein was incubated in various buffers at 4°C for 48 h and the remaining activity was measured at 50°C as described in Materials and Methods. The used buffers were listed as follows: pH 3.0–6.0, 100 mM sodium citrate buffer (black circles); pH 6.0–8.0, 200 mM sodium phosphate buffer (open circles); pH 8.6–10, 100 mM glycine-NaOH buffer (black triangle). Data were shown as means ± standard deviations from data in triplicate.

The sensitivity of EXLX1 to heat denaturation in aqueous solutions was assessed as the robustness of enzymes is an important factor for industrial applications. As shown in Fig.2a, EXLX1 was relatively stable when incubated at temperatures ranging from 25°C to 50°C for 30 min with more than 70% activities retained, while the residual synergistic activity decreased robustly when the temperature was higher than 50°C and completely disappeared at 70°C. The long-term thermal-stability of EXLX1 was also assessed due to the long reaction time required for lignocellulose hydrolysis of cellulase. The residual activity of EXLX1 declined significantly when the incubating time increased to 48 h and only about 50% of residual activity was retained after incubating at 50°C for 48 h (Fig.2a). Although temperature presented a significant effect on the activity of EXLX1, EXLX1 is still more thermal-stable than some previous reported expansins or expansin-like proteins [11], [23]. The creep activity of EXLX1 has been found to be high in the reaction buffer with pH from 5.5–9.5 [18]. Similarly, the EXLX1 pretreated at various buffers with pH ranging from 4.8–9.5 for 48 h (Fig.2b) was found to be active at the reaction buffer in this study (pH 4.8).

### Digestibility of UNT wheat straw with different cellulase and EXLX1 combinations

**Figure 3:**
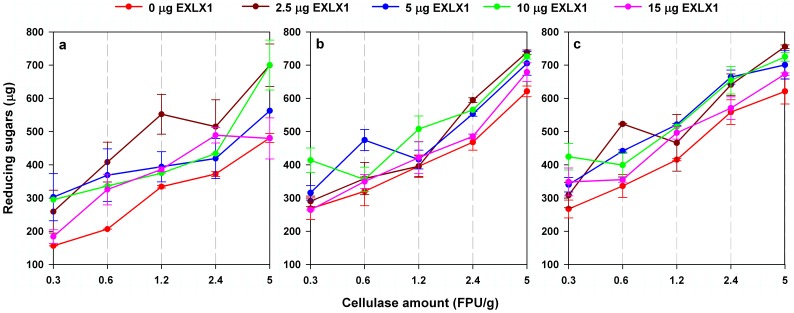
Reducing sugars (RS) released from UNT wheat straw using different combinations of cellulase and EXLX1 after incubation at 50°C for 24 h (a), 36 h (b) and 48 h (c) . The dosage of cellulase for hydrolysis ranged from 0.3–5 FPU/gds, while the EXLX1 ranged from 0–15 µg. Four replicates were performed for each determination. Data were shown as mean ± standard error.

**Figure 4:**
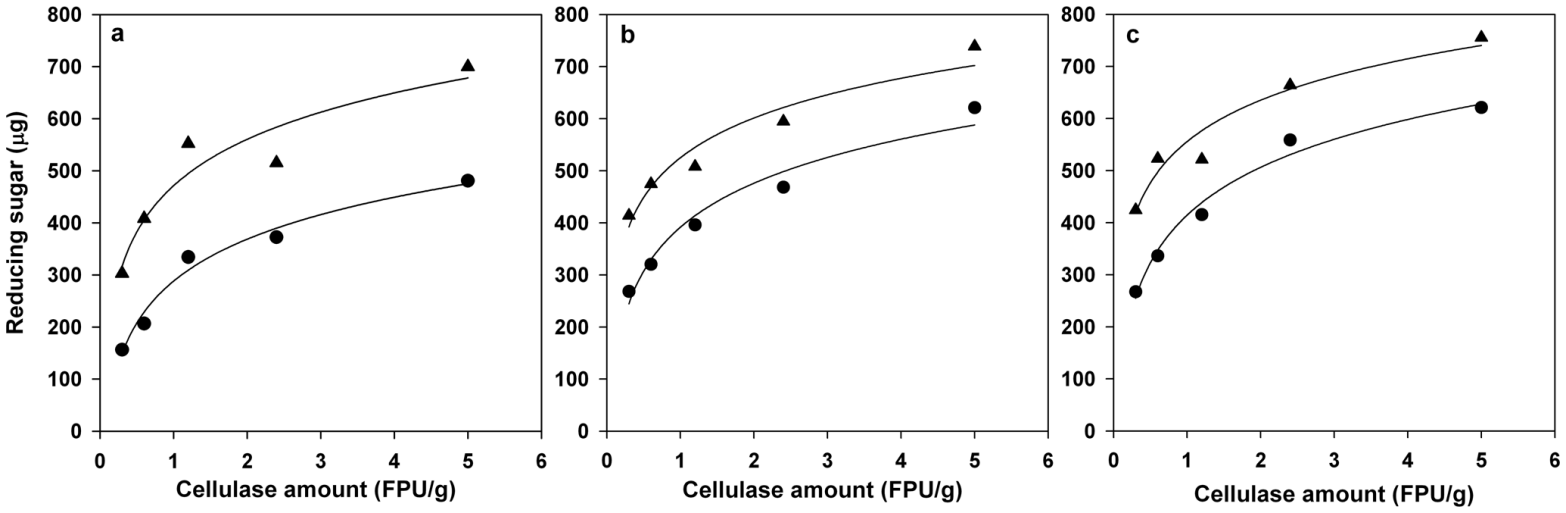
Comparison of reducing sugars (RS) yield of UNT wheat straw using optimized EXLX1 and cellulase combination (black triangles) and cellulase alone (lack circles), respectively. The hydrolysis was conducted at 50°C for 24 h (a), 36 h (b) and 48 h (c), respectively. Points and lines represented the mean values of four replicates and calculated values based on logarithmic equation (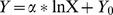), respectively. X is cellulase dosage (FPU/g). Y is RS yield. The estimated values of *α*, *Y_0_* and determination coefficient (*R^2^*) under specific conditions were listed as follows: Cellulase alone for 24 h, *α* = 116.26, *Y_0_* = 287.92, *R^2^* = 0.9775; Optimized cellulase and EXLX1 mixture for 24 h, *α* = 128.58, *Y_0_* = 471.14, *R^2^* = 0.9033; Cellulase alone for 36 h, *α* = 122.02, *Y_0_* = 391.46, *R^2^* = 0.9612; Optimized cellulase and EXLX1 mixture for 36 h, 110.10, *Y_0_* = 524.92, *R^2^* = 0.9385; Cellulase alone for 48 h, *α* = 132.54, *Y_0_* = 414.45, *R^2^* = 0.9813; Optimized cellulase and EXLX1 mixture for 48 h: *α* = 114.64, *Y_0_* = 555.74, *R^2^* = 0.9405.

Enzymatic hydrolysis of UNT wheat straw was performed by using different cellulase and EXLX1 combinations with the corresponding RS yields shown in Fig.3. ANOVA analysis was subsequently provided to study the effects of cellulase dosage (0.3–5 FPU/g), EXLX1 amount (0–15 µg) and hydrolysis time (24–48 h) on the synergism of EXLX1 in the hydrolysis process. It could be found that all the factors and their interactions exhibited significant (*p*<0.05) effects on the synergism with the order Time>Cellulase>EXLX1>EXLX1×Time>EXLX1×Cellulase>EXLX1×Time×Cellulase. The results suggested that the effect of EXLX1 amount on the synergism would be interfered by both cellulase and incubation time so that the effective synergism of EXLX1 with cellulase occurred only when a specific dosage of cellulase was used combined with an appropriate amount of EXLX1. The optimal amount of EXLX1 for each treatment with specific cellulase dosage and incubation time can be observed in Fig.3. RS yields obtained from the treatments using the optimal cellulase and EXLX1 combinations were sorted out and fitted with logarithmic equation (Fig.4). As shown in Fig.4, the growth rate of RS yield at 24 h brought by the increase in cellulase dosage was found to be higher in the treatment using an optimal cellulase and EXLX1 mixture than that using cellulase alone, indicating that the hydrolysis efficiency of the treatment using the cellulase and EXLX1 mixture responds more sensitively to the changes in the cellulase dosage than that using pure cellulase. However, the growth rate of RS yield decreased with the proceeding of the incubation time in the treatments using optimal cellulase and EXLX1 mixture but increased in those using pure cellulase (Fig.4).

Generally, all the treatments using cellulase alone released lower quantities of RS than that using the optimal mixture of cellulase and EXLX1, regardless of cellulase amount and incubation time (Fig.4). The increasing percentage of RS yield brought by changing the catalyst from pure cellulase to cellulase and EXLX1 mixture was even higher than that brought by increasing the cellulase dosage in some cases (Fig.4). For example, using 1.2 FPU/g of cellulase in combination with 2.5 µg of EXLX1 gave 552.26 µg of RS after 24 h incubation, while only 480.91 µg of RS released using 5 FPU/g of pure cellulase under the same condition. The digestion time could also be shortened after the addition of EXLX1. At the same cellulase loading, the RS yield obtained at 48 h using cellulase alone could be achieved at a shorter time owing to the synergism of EXLX1 (Fig.4). For example, 48 h of incubation was required for releasing 620.97 µg of RS when using 5 FPU/g of pure cellulase but only 24 h of incubation was required for releasing 699.77 µg of RS when using the mixture of 5 FPU/g cellulase and 5 µg EXLX1. All the results clearly demonstrated the enhancement effect of EXLX1 on cellulase activity using UNT wheat straw, which possesses more complexity than a pure cellulose fiber-like filter paper. According to the above-mentioned discussion, it could be suggested that EXLX1 might be an effective cellulase synergist to improve the RS released from lignocellulosic biomass wastes after appropriate optimization.

### Characteristics of EXLX1 for various lignocellulosic wastes with different pretreatments

#### General composition of extractives free lignocellulosic wastes

Various types of lignocellulosic wastes such as wheat straw, rice straw, switchgrass, reed leaves, reed stalk, sugarcane bagasse and wood were used as substrates. Two representative pretreatment methods (i.e. acid pretreatment and alkali pretreatment) were used in the present work. Table 1 listed the average composition (dry basis) of the extractives free biomass with or without pretreatment. Biomass samples used in this study, except of wood and reed stalk, contained a large amount of extractives about over 25% of the day matter (data not shown). The content of crude fibers including both cellulose and hemicellulose were higher in ALKALI materials than in ACID materials, while the lignin content exhibited the opposite (Table 1). Almost all the hemicellulose was completely removed after the acid pretreatment.

**Table 1:** Average composition (dry basis) of the biomass samples with or without pretreatment.

	Cellulose (%)			Hemicellulose (%)			Lignin (%)		
Biomass	1	2	3	1	2	3	1	2	3
A	64.52±1.50	69.66±0.23	69.51±1.73	19.35±0.12	2.25±0.36	18.29±0.34	16.13±1.62	28.09±2.08	12.20±2.07
B	63.64±3.28	44.44±7.57	74.42±1.98	25.00±0.13	7.78±1.55	19.77±2.20	11.36±3.15	47.78±5.12	5.81±2.40
C	56.16±1.19	67.93±1.62	74.12±1.87	30.14±0.74	0.90±0.12	14.12±0.48	13.70±1.93	31.18±3.18	11.76±2.35
D	20.59±0.92	66.22±4.65	64.10±1.06	73.53±2.42	4.06±0.69	25.64±0.34	5.88±3.34	29.73±4.03	10.26±0.44
E	53.09±1.26	68.54±1.62	70.79±3.32	30.86±0.38	3.38±0.63	20.22±2.55	16.05±1.64	28.09±1.67	8.99±0.75
F	52.31±3.88	61.29±8.73	55.70±3.79	24.62±1.54	5.38±0.38	22.78±0.97	23.08±5.42	33.33±4.84	21.52±4.77
G	55.81±0.35	57.29±0.95	64.33±1.23	11.63±0.31	5.21±1.56	0.68±0.04	32.56±0.27	37.50±1.65	34.99±1.27

1: UNT materials; 2: ACID materials; 3: ALKALI materials.

A: Switchgrass; B: Sugarcane bagasse; C: Wheat straw; D: Reed leaves; E: Reed stalk; F: Rice straw; G: Wood.

#### Conversion efficiencies

**Figure 5:**
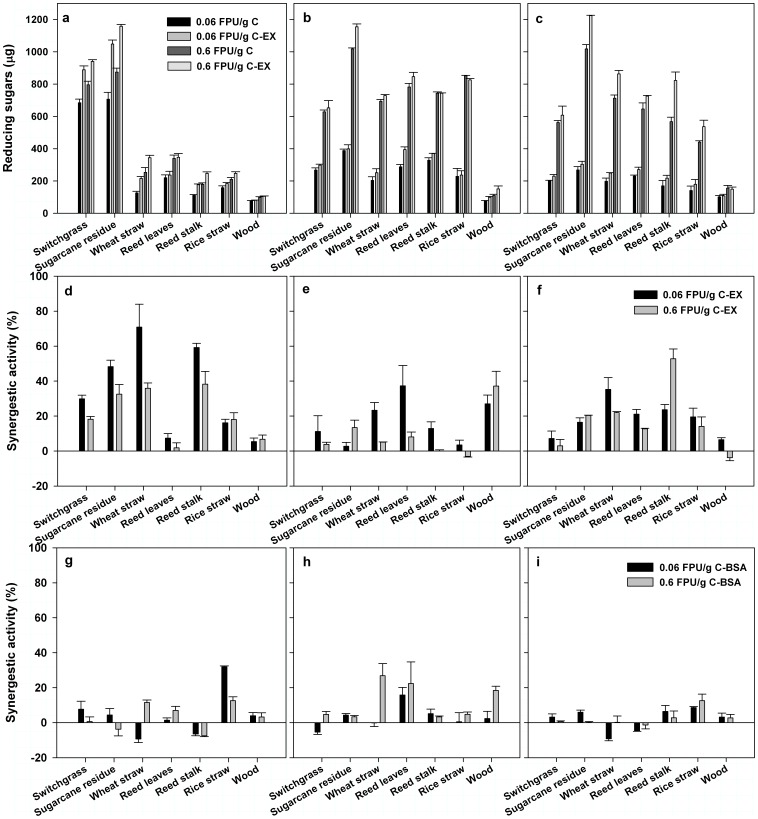
Hydrolysis activities of EXLX1 and cellulase combinations (a,b,c) and synergistic effects of EXLX1 (d,e,f) and BSA (g,h,i) on cellulase using various types of lignocellulosic biomass samples, including un-treated samples (a,d,g), dilute sulfuric acid pretreated samples (b,e,h) and alkali pretreated samples (c,f,i). The hydrolysis was conducted at 50°C for 36 h. 0.06 FPU/gds C: 0.06 FPU/gds of Cellulase; 0.6 FPU/gds C: 0.6 FPU/gds of Cellulase; 0.06 FPU/gds C-EX: 0.06 FPU/gds of Cellulase added with 0.5 µg EXLX1; 0.6 FPU/gds C-EX: 0.06 FPU/gds of Cellulase added with 5 µg EXLX1; 0.06 FPU/gds C-BSA: 0.06 FPU/gds of Cellulase added with 0.5 µg BSA; 0.6 FPU/gds C- BSA: 0.06 FPU/gds of Cellulase added with 5 µg BSA.

As shown in Fig.5a, the RS yielded from UNT switchgrass and UNT sugarcane bagasse after the hydrolysis of pure cellulase were higher than that from other UNT materials under the same condition. It is interesting to found that hydrolysis of these two UNT materials using a cellulase and EXLX1 combination resulted in a higher RS yield in comparison with that using cellulase alone, although the cellulase dosage in pure cellulase treatments were 9-fold higher than that in EXLX1 and cellulase mixture treatments (Fig.5a). Take UNT switchgrass treatments for example, the RS yield increased by 30.0% by using the cellulase (0.06 FPU/g) and EXLX1 mixture instead of pure cellulase (0.06 FPU/g), while the RS yield increased by only 16.2% by increasing the dosage of the pure cellulase from 0.06 FPU/g to 0.6 FPU/g. The above-mentioned phenomenon was also found in UNT sugarcane bagasse treatments. However, when other UNT materials such as rice straw, wheat straw, reed stalk, reed leaves and wood were used as substrates, increasing the cellulase dosage in reaction systems could be more effective than adding EXLX1 into cellulase as a synergist (Fig.5a). Chemical-physical pretreatment is known to be an important process in the real-world lignocelluloses saccharification that can improve the conversion efficiency of lignocelluloses in some enzymatic hydrolysis processes. In this work, remarkable enhancement in RS yields brought by pretreatments was observed in treatments using a majority of plant samples (Fig.5b and Fig.5c). However, when the substrate was sugarcane bagasse or switchgrass, both ACID and ALKALI pretreatment exerted negative effects on the final RS yields. It is obvious that the combined utilization of EXLX1 and cellulase was more efficient for improving the conversion efficiency of sugarcane bagasse and switchgrass in comparison with chemically pretreating the plant samples. Indeed, we found that UNT switchgrass and UNT sugarcane bagasse contained extractives in a high proportion (Table 1), which might contain free RS in a large content. During the pretreatment, free RS of extractives that affects the final RS yield was removed and finally resulted in the low RS yield after hydrolysis of the pretreated switchgrass and the pretreated sugarcane bagasse. In general, the enhancement induced by the synergistic effect of EXLX1 on the biomass conversion efficiency under some conditions would even higher than not only that induced by adding cellulase in a high amount but also that induced by chemically pretreating the plant samples before hydrolysis, although the conversion efficiency of the saccharification system exhibited a negative effect on the synergistic capacities of EXLX1 in most cases [6], [16].

#### Synergistic characteristics of EXLX1

Synergistic activities of EXLX1 on cellulase for various biomass samples with different pretreatments were shown in Fig.5d, Fig.5e and Fig.5f. Negative control experiments using BSA instead of EXLX1 were also conducted with results shown in Fig.5g, Fig.5h and Fig.5i. Comparison of the synergism of EXLX1 and that of BSA showed that the effects of BSA on the cellulase activity in the hydrolysis process were completely different from that of EXLX1. For example, the higher synergism was more likely to appear in the BSA treatments with a higher amount of BSA but in the EXLX1 treatments with a lower amount of EXLX1. Besides, although BSA exhibited significantly positive effects on the cellulase activity in some cases, the average synergistic activity of EXLX1 was higher than that of BSA. All the above-mentioned results demonstrated that the synergistic effects observed in this work was specific to EXLX1. Indeed, the specific effect of EXLX1 or some other expansin-like proteins on glycoside hydrolase (e.g. cellulase, xylanase) has also been proposed by other researchers [6], [13], [24].

EXLX1 presented different synergisms for different lignocellulose biomass substrates (Fig.5d–f), but may be governed by the biomass composition. According to the results in Table 1, most of UNT materials contained more than 50% of cellulose without remarkable differences in the proportion except for UNT reed leaves. Insignificantly low correlations (0.06 FPU/g cellulase: Pearson Correlation  = 0.055, *p* = 0.918; 0.6 FPU/g cellulase: Pearson Correlation  = −0.024, *p* = 0.964) were observed between the synergistic activity and the cellulose proportion of materials, when the synergistic activity data and the cellulose proportion data from all the treatments except for UNT reed leaves treatments were pooled for correlation analysis. Despite it, the cellulose proportion of materials may be an important factor affecting the synergistic activity of EXLX1, since EXLX1 exhibited a low synergism when UNT reed leaves that contain an extremely low proportion of cellulose were used as substrates. Correlation analysis of the synergistic activity of EXLX1 and the hemicellulose proportion was also performed using the pooled data from the treatments excluding UNT reed leaves treatments. A significantly positive relationship was found between the synergistic activity and the hemicellulose proportion (0.06 FPU/g cellulase: Pearson Correlation = 0.857, *p*<0.05; 0.6 FPU/g cellulase: Pearson Correlation = 0.930, *p*<0.05). Besides, correlation analysis results showed that the synergistic activity correlated negatively with the lignin content (0.06 FPU/g cellulase: Pearson Correlation = −0.829, *p*<0.05; 0.6 FPU/g cellulase: Pearson Correlation = −0.845, *p*<0.05). Indeed, the average synergistic activity (0.06 FPU/g cellulase: 18.52%; 0.6 FPU/g cellulase: 18.48%) of EXLX1 using ALKALI materials containing crude fibers in a high proportion and lignin in a low proportion was found to higher than that (0.06 FPU/g cellulase: 16.86%; 0.6 FPU/g cellulase: 9.24%) using ACID materials containing lignin in a high proportion. The results indicated that a higher synergism of EXLX1 is prone to appear in the treatment with the substrate that contains a high proportion of hemicellulose and a low proportion of lignin, when the cellulose proportion of different biomass materials exhibited no remarkable differences. The information gained in the present study would be favorable to assess whether EXLX1 is effective as a cellulase synergist for the hydrolysis of the determined lignocellulosic biomass. Although the mechanism for the relationship between the lignocellulose composition and the synergism effect of EXLX1 is still unknown, we supposed that it might be linked to the unique feature of expansin in breaking hydrogen bonds between hemicellulose and cellulose, which has been suspected of facilitating the cellulase targeting of the wall polysaccharide network [25], [26].

#### Binding characteristics of EXLX1

The binding capacity of EXLX1 is known to affect its wall extension activity [18]. As shown in Table 2, the binding affinity of EXLX1 to different biomass samples was significantly different. EXLX1 displayed much higher binding to UNT wheat straw and UNT rice straw than to other UNT materials, while no adsorption of EXLX1 to UNT reed leaves was found. The binding affinity of EXLX1 to different biomass samples might be related to the lignocellulosic components as the UNT reed leaves contained an extremely lower proportion of cellulose than other UNT materials (Table 1). EXLX1 bound to all the ACID and ALKALI biomass samples and exhibited much higher binding to ALKALI materials than to ACID materials at 30°C in the acidic reaction buffer with pH 4.8, regardless of biomass types (Table 2). Similar results were also found at 30°C in the neutral reaction buffer with pH 7.0 (Table 3). It has been suggested that lignin is nonspecifically bind with EXLX1 [15], while cellulose specifically binds with this protein and is controlled by the D2 domain of EXLX1 with hydrophobic amino acid residues responsible for binding cellulose [18], [25]. In this work, EXLX1 exhibited more preferential binding for ALKALI materials at 30°C than ACID materials, indicating that the binding activity of EXLX1 to polysaccharide components might be more active than that to lignin under the assay conditions. Kim et al [15], however, proposed that EXLX1 has preferential binding for *M. xgiganteu* after alkali pretreatment than that after acid pretreatment at 4°C. The variability may be due to that many factors would affect the binding behavior of EXLX1 [14], [27]. For example, the plant biomass wastes generally contained a high content of chemicals involved in the soluble extractives such as divalent cations and NaCl, which would affect the binding affinity of EXLX1 [27]. Besides, it was further found that the quantities of EXLX1 bound to ACID reed stalk and ACID sugarcane bagasse exceeded the amounts bound to the corresponding ALKALI samples when the temperature was set at 4°C (Table 3). The result suggested that the binding affinity of EXLX1 might also be affected by incubation temperature.

**Table 2:** Binding to various lignocellulosic biomass samples with different pretreatments.

	Relative amount of bound EXLX1 (%)		
Biomass	UNT	ACID	ALKALI
Switchgrass	28.36±5.40	25.69±3.19	64.29±5.85
Sugarcane bagasse	33.59±2.71	69.23±4.72	91.13±3.63
Wheat straw	100.43±0.29	58.69±2.47	83.47±2.46
Reed leaves	−8.07±0.35	24.69±2.40	46.42±3.98
Reed stalk	20.43±4.91	21.91±3.43	48.58±0.45
Rice straw	70.48±1.88	50.78±3.12	65.39±3.32
Wood	23.40±5.75	20.18±0.91	54.37±2.70

Incubation was performed in citric buffer (50 mM, pH 4.8) at 30°C for 4 h.

**Table 3:** The binding preference study of EXLX1 with ALKALI materials and ACID materials as substrate, respectively.

	ALKALI binding preference (%) a	
Biomass	30°C	4°C
Switchgrass	25.86±1.45	12.20±0.31
Sugarcane bagasse	12.20±6.33	−37.47±2.39
Wheat straw	20.00±2.91	4.41±1.07
Reed leaves	3.67±0.56	−2.65±1.32
Reed stalk	33.06±0.31	−21.90±0.52
Rice straw	20.57±2.70	11.17±1.03
Wood	35.41±2.81	26.30±0.52

Incubation was performed in potassium phosphate buffer (50 mM, pH 7) at 4°C and 30°C for 4 h, respectively.

a Binding preference (%)  =  The relative amount of the bound protein toward ALKALI materials (%)-The relative mount of the bound protein toward ACID materials (%).

## Conclusions

As a kind of expansin can be actively produced in a large amount and purified effectively, EXLX1 has the promising potentiality as a cellulase synergist to improve the RS yielded from lignocellulosic wastes after optimization. The potential of EXLX1 as a cellulase synergist was related to lignocellulosic substrate type and would be governed by their composition. A higher synergism of EXLX1 with cellulase is prone to appear in the treatment using a material that contains a high proportion of hemicellulose and a low proportion of lignin when the cellulose proportion of different biomass materials exhibited no remarkable differences.

## Supporting Information

Table S1 Amino acid sequence of EXLX1.
(DOC)
