# Supplementary material for: Evaluation of Bacterial Expansin EXLX1 as a Cellulase Synergist for the Saccharification of Lignocellulosic Agro-Industrial Wastes
Source: PLoS One. 2013 Sep 23;8(9):e75022. doi: 10.1371/journal.pone.0075022 (PMC3781039; doi:10.1371/journal.pone.0075022)
Supplement: Table S1 — Amino acid sequence of EXLX1. (DOC) [file pone.0075022.s001.doc]

**Supporting information**

**Evaluation of bacterial expansin EXLX1 as a cellulase synergist for the saccharification of lignocellulosic agro-industrial wastes**

Hui Lin ^1^, Qi Shen ^1^, Ju-Mei Zhan ^2^, Qun Wang ^1^, Yu-Hua Zhao 1[[1]](#footnote-2)*

^1^ Institute of Microbiology, College of Life Sciences, Zhejiang University, Hangzhou 310058, People’s Republic of China.

^2^ Institute of Plant Science, College of Life Sciences, Zhejiang University, Hangzhou 310058, People’s Republic of China.

**Table S1** Amino acid sequence of EXLX1

| Protein | Amino acid sequence |
| --- | --- |
| EXLX1 | N’-MASMTGGQQMGRGSEFAYDDLHEGYATYTGSGYSGGAFLLDPIPSDMEITAINPADLNYGGVKAALAGSYLEVEGPKGKTTVYVTDLYPEGARGALDLSPNAFRKIGNMKDGKINIKWRVVKAPITGNFTYRIKEGSSRWWAAIQVRNHKYPVMKMEYEKDGKWINMEKMDYNHFVSTNLGTGSLKVRMTDIRGKVVKDTIPKLPESGTSKAYTVPGHVQFPELEHHHHHH-C' |

1. ^^ Corresponding author. Tel:+86-571-88208557, fax: +86-571-88206995

   E-mail addresses: [yhzhao225@zju.edu.cn](mailto:yhzhao225@zju.edu.cn) (Y. Zhao) [↑](#footnote-ref-2)
